# Sports participation, perceived neighborhood safety, and individual cognitions: how do they interact?

**DOI:** 10.1186/1479-5868-8-76

**Published:** 2011-07-21

**Authors:** Mariëlle A Beenackers, Carlijn BM Kamphuis, Alex Burdorf, Johan P Mackenbach, Frank J van Lenthe

**Affiliations:** 1 Department of Public Health, Erasmus University Medical Center, PO Box 2040, 3000 CA Rotterdam, the Netherlands

**Keywords:** physical activity, sport, safety, moderator variable, environment

## Abstract

**Background:**

Little is known about the interaction between individual and environmental determinants of physical activity, although this may be important information for the development of effective interventions. The goal of this paper is to investigate whether perceived neighborhood safety modifies associations between individual cognitions and sports participation.

**Methods:**

Cross-sectional data were obtained from residents (age 25-75) of 87 neighborhoods in the city of Eindhoven, who participated in the Dutch GLOBE study in 2004 (N = 2474). We used multilevel logistic regression to analyze the interactions between perceived neighborhood safety and individual cognitions (attitude, self-efficacy, social influence, and intention) on sports participation (yes/no).

**Results:**

In its association with sports participation, perceived neighborhood safety interacted significantly with self-efficacy and attitude (p < 0.05). Among persons who perceived their neighborhood as safe, a positive attitude was strongly associated with sports participation (OR = 2.00, 95%CI = 1.48-2.71). In contrast, attitude was not associated with sports participation in persons who perceived their neighborhood as unsafe (OR = 0.65, 95%CI = 0.34-1.24). Further, self-efficacy was significantly stronger associated with sports participation in persons who perceived their neighborhood as unsafe (OR = 1.85, 95%CI = 1.31-2.60) than in those who perceived their neighborhood as safe (OR = 1.19, 95%CI = 1.05-1.36). Social influence and intention did not interact with perceived neighborhood safety.

**Conclusions:**

Associations between individual cognitions and sports participation depend on neighborhood circumstances, such as perceived neighborhood safety. Interventions to promote sports participation in adults should take the interaction between environmental and individual characteristics into account. More research is needed to find out the causal pathways in individual-environment interactions.

## Background

Regular physical activity (PA) prevents major chronic diseases such as diabetes, cardiovascular disease, mental illness, obesity, and various types of cancer [1,2]. Although the health benefits of regular exercise and a physically active lifestyle are well known, many people are still not active. In the Dutch population, over 40% does not meet the national recommendation of being moderately active for at least half an hour on at least five days a week [3,4]. In the US, the percentage of people not reaching the recommended level of PA is over 50% [5]. Therefore, increasing PA comprises a large potential public health gain [1,6].

Previously, the promotion of PA has focused mainly on changing individual cognitions towards PA, such as attitude and self-efficacy [7,8]. Over the past decade, the focus of research has shifted more to environmental determinants of health and health behavior [9]. In addition, ecological models suggest that health behavior is determined by individual as well as environmental factors and that they are interrelated [10,11]. So far, little is known about these individual-environment interactions.

Sports participation is an important element of PA. Persons who participate in sports have a lower mortality than those who do not participate in sports [12]. In Europe, only 40% of the adult population participates in sports with some regularity, ranging from 72% in Finland, to only 13% in Bulgaria [13]. In the US, 24% of the population is regularly vigorously physically active [5]. An environmental factor that has been suggested to be related to PA and sports participation is neighborhood safety [14,15]. In the US, higher levels of perceived neighborhood safety were associated with lower levels of physical inactivity [16]. A study by McGinn and colleagues reported that both perceived as objectively measured crime were related to physical activity [17].

Why does neighborhood safety influence physical activity? Macintyre suggests that the importance of environmental factors related to health are roughly following the order of human needs as defined by Maslow [18,19]. In this order of human needs, safety is one of the main needs, just after air, water, food, and shelter [19]. When a basic need like safety is unfulfilled, higher ranked needs, like sport participation, are less relevant.

Another explanation for the association between neighborhood safety and physical activity is that people most often have to leave their house when they want to exercise. An unsafe environment might act as a barrier for sports participation. Especially since, in the Netherlands, adults are most involved in sports activities in the evenings and weekends due to other responsibilities during the day. For types of sports that start from the doorstep (like running and cycling), this association is rather obvious, as these sports completely or partly take place in the neighborhood. For sports that are played at a sports club outside the own neighborhood, neighborhood safety may also act as an important perceived barrier, as one has to travel through his or her own neighborhood to get there.

A large pan-European study showed that perception of safety was associated with an increase in the likelihood to engage in occasional exercise of 22% in women and 39% in men [20]. Sallis and colleagues [21] showed that women who reported low levels of crime in their neighborhood reported about an hour more moderate and vigorous physical activity compared to women who reported high levels of crime in their neighborhood. In a previous study by Kamphuis et al [22], it was demonstrated that people who perceived their neighborhood as safe were almost twice as likely to participate in sports than those who perceived their neighborhood as unsafe.

However, not all studies find a positive association between perceived safety and PA [23,24]. Whether perceived neighborhood safety is a barrier for sports participation is likely to depend on individual cognitions. It seems plausible that positive cognitions towards PA might help people to deal with environmental barriers. The exact nature of this interdependency is largely unknown. Although previous studies have focused on the association between perceived neighborhood safety or individual cognitions and sports participation, very few investigated their interaction. For example, Deforche and colleagues [25] found that feelings of unsafety were only associated with the likelihood of active transportation in youth who had low self-efficacy and not in youth who had a strong self-efficacy. Thus, the aim of this study is to investigate whether perceived neighborhood safety modifies the associations between individual cognitions and sports participation.

## Methods

### Study population

Data were obtained in a large-scale postal survey, a component of the most recent wave of data collection for the longitudinal Dutch GLOBE study (October 2004). The cross-sectional data originated from a stratified sample of the adult population of Eindhoven and its surrounding municipalities (N = 4785; response 64.4%). More detailed information on the objectives, study design, and data collection of the Dutch GLOBE study can be found elsewhere [26,27]. The use of personal data in the GLOBE study is in compliance with the Dutch Personal Data Protection Act and the Municipal Database Act, and has been registered with the Dutch Data Protection Authority (number 1248943).

Since we suspect that safety concerns are different in a city environment compared to a rural environment, only participants residing in the city of Eindhoven (N = 2917) were selected. Eindhoven is the fifth largest city in the Netherlands with over 200,000 inhabitants. Respondents lived spread throughout the whole city. Individuals with missing data on the outcome measure or on one of the confounding variables, i.e. age, sex, education, or country of origin, were omitted (N = 356). Respondents who had missing values on more than 25% of the items of individual cognition and neighborhood safety were also omitted (N = 87). A total number of 2474 respondents were analyzed. These respondents resided in 87 of Eindhoven's administrative neighborhoods (mean number of respondents per neighborhood N = 28, range 1 to 103).

### Measures

All measures used in this study were derived from self-reported data from the GLOBE postal survey of 2004.

#### Sports participation

Sports participation was measured using the SQUASH questionnaire, which is a validated questionnaire for measuring different types of PA among an adult population [28]. Respondents could record up to four different sport activities they had done in an average week over the past few months (open question, no defined list given). For each sport activity, they had to report the frequency (times per week), the average duration (minutes per day) and the intensity (low, average, high). In combination with the respondent's age and the activity-specific metabolic equivalent of task (MET) values, the self-reported intensity was used to calculate intensity scores. The total number of minutes per week with at least moderate intensity (moderate intensity = 4-6 MET for 18-55 yrs-old; 3-5 MET for 55+ yrs-old) was calculated. Since about half of the respondents did not do any sports, sports participation was dichotomized into 'yes' for respondents who participated in sports with moderate or high intensity at least once a week for at least 30 minutes versus 'no' for those who did not participate in sports weekly.

#### Individual cognitions

The individual cognition items were formulated as individual cognitions towards 'sufficient PA' (see Additional File 1, Table S1). The cognitions used in this study were derived from commonly employed health behavior theories such as the Social Cognitive Theory and the Theory of Planned Behavior [7,8]. Attitude (eleven items, Cronbach's alpha = .77), self-efficacy (two items, Cronbach's alpha = .75), and intention (one item) were measured on a five-point ordinal scale, and social influence (three items, Cronbach's alpha = .72) was measured on a three-point ordinal scale. The percentage of missing observations varied between 1.7% and 4.9% for the items for attitude, self-efficacy and social influence, while there were 9.5% missing observations for the item 'intention'. Missing values were imputed by using the expectation maximization (EM) algorithm [29] from SPSS version 15.0. For all individual cognitions (except intention) a mean score was calculated from the relevant items within each cognition. A higher score on each of the individual cognition scales represented a more positive cognition. Individual cognitions were mean-centered for analytical purposes. All individual cognitions were treated in the analyses as continuous variables.

#### Perceived safety of the neighborhood

Perceived safety of the neighborhood was assessed with four items. The first three items assessed people's fear of being home alone or of going out on the streets in their neighborhood in the daytime or at night. The items were dichotomized into 'no, never feeling afraid' (0) and 'neutral/yes, sometimes feeling afraid' (1). The fourth item asked the respondents whether they thought their neighborhood was unsafe (no = 0, yes = 1). These four dichotomous items were summed up to form a scale (Cronbach's alpha = .67).

The first three items about fear had just over one percent (1.3-1.4%) missing observations. These missing values were imputed using the EM algorithm. The fourth item about neighborhood safety had 5.9% missing observations. The missing values of this (dichotomous) item were imputed using the predicted group membership from a logistic regression with the other three safety items and several social disorganization items from the survey as predictor variables ("How frequent do the following adverse events occur in your neighborhood?" Items referred to examples such as litter, graffiti, vandalism, and violence.).

Respondents who did not agree with any of the items indicating an unsafe neighborhood were regarded as 'high' on perceived neighborhood safety. Respondents who agreed once or twice to a measure indicating an unsafe neighborhood were considered 'medium' on perceived neighborhood safety. Respondents who agreed to three or four of the items indicative of an unsafe surrounding were considered 'low' on perceived neighborhood safety.

#### Demographics

Possible confounders were age, sex, country of origin (the Netherlands, other country), and educational level ((1) no education or primary education; (2) lower professional and intermediate general education; (3) intermediate professional and higher general education; (4) higher professional education and university). Educational level was included as an indicator for socio-economic status (SES) and has proven to be a good measure for SES in the Netherlands [30].

### Statistical analyses

Crude and multivariable logistic regressions were used to explore the associations between individual cognitions and sport participation, and between perceived neighborhood safety and sport participation. All multivariable models were adjusted for age, sex, educational level, and country of origin. To assess interactions between individual cognitions and perceived neighborhood safety, a backward logistic regression was performed in which all possible interaction terms between perceived neighborhood safety and the individual cognitions were included. These analyses were carried out in SPSS version 15.0.

Because of the hierarchical structure of the data, a multilevel analyses was performed using MLwiN (version 2.02) using the logit-link function and 2^nd ^order PQL estimation methods [31]. In the multilevel models, all the significant variables (p < 0.05) from the crude analyses (model 1) and all the significant interactions (p < 0.05) from the backward logistic regression (model 2) were included.

Parameters in logistic regression models that include an interaction are difficult to interpret. To clarify this, a simplified interactive logistic regression model (equation 1) was formulated which was reduced to only one quantitative variable (X), one categorical variable with three levels (Z), and the interaction between these two variables (XZ). In this study, X represents an individual cognition (e.g. attitude) and Z represents perceived neighborhood safety with three levels: high, medium, and low.(1)

In this equation, P is the probability of participating in sports, α is the constant and β_1 _is the coefficient that reflects how much the log odds will change when the individual cognition increases with one unit. However, because of the interaction term in the model, the association of X on the outcome is conditional on the reference level of perceived neighborhood safety (Z_high_) (equation 2). In other words, the coefficient of the interaction term should be interpreted as a multiplicative factor.

To obtain the coefficient of the individual cognition (X) for the second category of perceived neighborhood safety (Z_medium_), the coefficient of X (β_1_) should be multiplied by the coefficient of the interaction term XZ_medium _(β_6_) (equation 3).

To obtain the coefficient of the individual cognition (X) for the last category of perceived neighborhood safety (Z_low_), the coefficient of X (β_1_) should be multiplied by the coefficient of the interaction term XZ_low _(β_7_) (equation 4).

The other coefficients of the variables that are part of the interaction term should also be interpreted carefully. Because Z_high _is the reference category, its value is zero. Therefore, coefficients β_2 _and β_5 _are zero. The coefficients β_3 _and β_4 _are the coefficients for the medium and low levels of perceived neighborhood safety, which are conditional on the 0-value of the individual cognition (X). Since the individual cognitions were mean-centered, the coefficients can be interpreted as the typical effect of the perceived neighborhood safety when the individual cognition is at its mean.

The analyses were carried out for both the imputed and non-imputed datasets and they provided similar results. We present the data of the imputed dataset.

## Results

Table 1 shows the characteristics of the sample. Almost half of the sample participated in sports with moderate or high intensity at least once a week for at least 30 minutes.

**Table 1:** Characteristics of the GLOBE study respondents living in the city of Eindhoven.

	Sample^a^	
	**N**	**%**

**Total sample**	2474	100
**Sex**		
Male	1168	47.2
Female	1306	52.8
**Age **mean (range)	53.1 (25-75)	
25-34	340	13.7
35-44	409	16.5
45-54	413	16.7
55-64	668	27.0
65-75	644	26.0
**Education**		
1 Low	243	9.8
2	890	36.0
3	571	23.1
4 High	770	31.1
**Country of birth**		
Netherlands	2253	91.1
Other	221	8.9
**Sports participation**		
Yes	1308	47.1
No	1166	52.9

a. The numbers and percentages presented are unweighted and are therefore a representation of the actual numbers in the dataset.

In the crude and the adjusted models, all individual cognitions were strongly positively associated with sports participation (table 2). Those who perceived their neighborhood as safe were twice as likely to participate in sports as those perceiving their neighborhood as unsafe. The associations remained similar when adjusted for age, sex, education, and country of origin.

**Table 2:** Crude and adjusted logistic regression analyses for sports participation.

		Crude		Adjusted^a^	
**Variables**		**OR^b^**	**95% CI**	**OR^a, b^**	**95% CI^a^**

**Individual cognitions**	**Mean (SD)**				
Attitude (1-5)	3.76 (0.54)	3.71 ***	**3.12-4.40**	3.50 ***	**2.94-4.18**
Self-efficacy (1-5)	3.82 (0.91)	1.92 ***	**1.74-2.11**	1.91 ***	**1.72-2.11**
Social influence (1-3)	2.28 (0.59)	1.63 ***	**1.42-1.87**	1.63 ***	**1.41-1.88**
Intention (1-5)	4.04 (1.02)	2.20 ***	**2.01-2.42**	2.10 ***	**1.91-2.31**
**Perceived neighborhood safety**	**%**				
Safety high (safe)	60.6%	1.00		1.00	
Safety medium	31.8%	0.75 **	**0.63-0.89**	0.81 *	**0.67-0.98**
Safety low (unsafe)	7.6%	0.36 ***	**0.26-0.50**	0.45 ***	**0.32-0.64**

a. Models were adjusted for age, sex, educational level and country of origin.

b. * = p < .050, ** = p < .010, *** = p < .001

In the multivariable model without interactions (model 1, table 3), attitude and intention were the strongest predictors of sports participation. When attitude increased by one unit (on a 5-unit scale), the odds of participating in sports increased by approximately 60% relative to the odds when attitude was at its mean value. When intention increased by one unit (on a 5-unit scale), the likelihood of sports participation increased by just over 50% relative to the odds when intention was at its mean value.

**Table 3:** Multilevel multivariable logistic regression models with OR and 95% CI for sports participation.

	Model 1^a^		Model 2^a^	
				
Variables	OR^b^	95% CI	OR^b^	95% CI
**Perceived neighborhood safety**				
Safety high (safe)	1.00		1.00	
Safety medium	0.90	0.74-1.09	0.90	0.74-1.09
Safety low (unsafe)	0.60 **	**0.43-0.84**	0.57 ***	**0.42-0.77**
**Individual cognitions**				
Attitude (1-5)^c^	1.60 ***	**1.27-2.01**	2.00 ***^d^	**1.48-2.71**
Self-efficacy (1-5)^c^	1.25 ***	**1.13-1.39**	1.19 **^d^	**1.05-1.36**
Social influence (1-3)^c^	1.24 **	**1.07-1.43**	1.25 **	**1.08-1.44**
Intention (1-5)^c^	1.51 ***	**1.35-1.68**	1.51 ***	**1.35-1.69**
**Interactions**				
Safety * attitude				
Safety high * attitude			1.00^e^	
Safety medium * attitude			0.69^e^	0.44-1.07
Safety low * attitude			0.33 ***^e^	**0.17-0.63**
Safety * self-efficacy				
Safety high * self-efficacy			1.00^e^	
Safety medium * self-efficacy			1.03^e^	0.79-1.33
Safety low * self-efficacy			1.55 *^e^	**1.07-2.24**

a. Models were adjusted for age, sex, educational level and country of origin

b. * = p < .050, ** = p < .010, *** = p < .001

c. The individual cognitions were centered around it's mean for analytical and interpretational purposes.

d. The OR of attitude and self-efficacy in model 2 represent the ORs of these two variables in a neighborhood perceived as safe (the reference category).

e. The parameters of the interaction terms should be interpreted as multiplicative factors. E.g.: to obtain the OR for self-efficacy for people who perceive their neighborhood as unsafe, one has to multiply the OR for the relevant interaction term (OR = 1.55) with the OR of self-efficacy (OR = 1.19). The calculated ORs for attitude and self-efficacy for each of the safety categories can be found in figure 1. More information on the interpretation of these parameters can be found in the method section.

Multilevel multivariable analyses showed significant interactions between attitude and perceived neighborhood safety and between self-efficacy and perceived neighborhood safety (model 2, table 3). Social influence and intention did not interact with perceived neighborhood safety.

These interactions are visualized in Figure 1. It shows that among persons who perceived their neighborhood as safe, a positive attitude increased the likelihood of sports participation (OR = 2.00, 95%CI = 1.48-2.71). The association between attitude and sports participation became weaker when the neighborhood was perceived as less safe. Among those who perceived their neighborhood to be unsafe, the association with attitude was no longer significant (OR = 0.65, 95%CI = 0.34-1.24). For self-efficacy, the interaction was the other way around: a strong self-efficacy increased the probability of sports participation significantly more in persons who perceived their neighborhood as unsafe (OR = 1.85, 95%CI = 1.31-2.60) relative to those who perceived their neighborhood as safe (OR = 1.19, 95%CI = 1.05-1.36).

**Figure 1:**
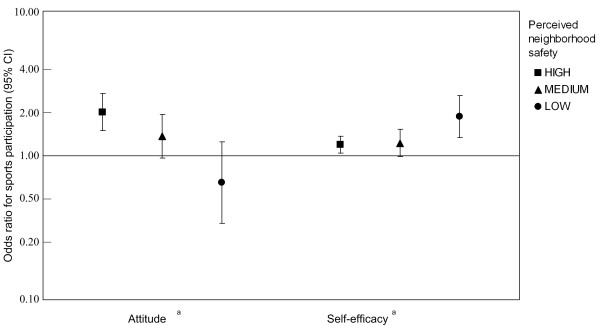
**OR and 95% CI for attitude and self-efficacy for three levels of perceived neighborhood safety**. The ORs were calculated by multiplying the OR of the individual cognition by the OR of the relevant interaction term (both derived from model 2 in table 3 which is adjusted for age, sex, educational level, country of origin, and all other individual cognitions).

## Discussion

This study is among the first to explore environment-individual interactions in sports participation. It showed that perceived neighborhood safety moderated the associations between attitude and sports participation, and between self-efficacy and sports participation. The associations between social influence and sports participation, and between intention and sports participation did not differ according to perceived neighborhood safety.

Similar with many other studies [32], attitude, self-efficacy, social influence, and intention were all important correlates of sports participation in this study. Our finding that perceived neighborhood safety was strongly associated with the likelihood of sports participation is in line with some, though not all studies [16,20,24,33]. To check whether this relationship was different for different types of sports, we compared respondents who participated in organized sports like tennis and basketball with non-participators, and respondents who participated in more "neighborhood oriented" sports like cycling, jogging, and walking with non-participators (results not shown). Although the association between perceived neighborhood safety and sports participation was stronger in those who participated in "neighborhood oriented" sports, the association was also significant for those participating in organized sports. This strengthens the assumption that perceived neighborhood safety might be an important factor for all sports participation either because the activity is carried out within the neighborhood or because people have to travel through their neighborhood. The interaction found in this study indicated that associations of self-efficacy and attitude with sports participation were modified by the environmental barrier of an unsafe neighborhood environment; where a strong self-efficacy may help people to overcome this barrier, having a positive attitude may not be enough to participate in sports when living in an unsafe neighborhood. In a safely perceived environment, on the other hand, attitude was more important for explaining sports participation than self-efficacy, since a strong self-efficacy may be less relevant for this situation. Similar to our study, Deforche and colleagues [25] also looked at the interaction between perceived safety and self-efficacy and found that, perceived safety was associated with active transportation in youth with low-self-efficacy only.

Since this study is cross-sectional, the interactions as observed can also be interpreted differently, that is, that individual cognitions moderate the association between perceived neighborhood safety and sports participation. In this interpretation, sports participation of those who have a strong self-efficacy is possibly less influenced by an unsafe environment. On the other hand, people who have a positive attitude might be more inhibited by an unsafe environment compared to those who have a negative attitude. This difference could be explained by the different nature of the two cognitions. A positive attitude is more related to whether someone *wants *to be physically active, while a strong self-efficacy is more related to whether someone feels he *can *be active. When someone *wants *to be active, but lives in an unsafe environment, he or she could perceive this as a barrier to become active. When someone has a negative attitude, and therefore, does not *want *to be active, he or she might also be less likely to perceive any barriers.

### Methodological considerations

An important limitation is the cross-sectional design. Therefore, no conclusions about causalities or the direction of the interactions can be drawn; the investigated associations of individual cognitions and neighborhood factors with sports participation can be bi-directional. The neighborhood can influence whether someone participates in sports, but, just as likely, participating in sports may influence the way people perceive their neighborhoods; as by participating in sports or travelling to the sports facility, they get exposed to their neighborhood. The same counts for individual cognitions. A mechanism that may be involved in this process is 'cognitive dissonance' [34], which describes the cognitive process in which people adjust their beliefs to match their actions; persons who are not active may adjust their cognitions or even their perceptions of the neighborhood to match their behavior. The interactions can also be interpreted both ways: It can be interpreted as if the perceived neighborhood safety moderates the associations between cognitions and sports participation, but another explanation could be that the cognitions moderate the association between perceived neighborhood safety and sports participation.

When interpreting the results, one should be aware that only perceptions about the safety of the neighborhood are considered in this study. From the results we can infer that feeling unsafe in the neighborhood is associated with a lower probability of sports participation. However, we cannot determine why people are feeling unsafe because this was not stated in the question posed. Another reason is that there are many factors, apart from the real safety in a neighborhood, which can affect perceived neighborhood safety [24]. It would therefore be interesting to see if these interactions can also be found in a study that includes objective measures of neighborhood safety.

Moreover, self-reported data were used, which may have led to an over-reporting of PA [35,36] or an overestimation of strength of associations between determinants and sports participation due to same-source bias. Lastly, individual cognitions were not measured specifically regarding sports participation but for PA in general.

### Implications for research and practice

This study is a first exploration of interactions between individual and environmental correlates of sports participation and it suggests that these are important for understanding health behavior. Further research should incorporate both objective and subjective measures of safety when investigating interactions regarding PA behaviors. Moreover, studies need to explore interactions with other important environmental determinants such as neighborhood aesthetics. Although cross-sectional designs are helpful in exploring the possible relations, stronger designs are needed to confirm causal pathways. It is also important to explore interactions for other types of health behaviors.

This study implies that when developing interventions to promote PA, the specific individual cognitions that should be targeted may differ by how persons perceive their neighborhood. It may also imply that whether an improvement of neighborhood safety results in more sports participation depends on the specific individual cognitions people hold.

## Conclusion

Associations between individual cognitions and sport participation depend on neighborhood circumstances such as perceived neighborhood safety. More research is needed to find out the causal pathways in individual-environment interactions with regard to health behaviors.

## Competing interests

The authors declare that they have no competing interests.

## Authors' contributions

MAB carried out the data-analyses and drafted the manuscript. CBMK collected the data described in this study and critically commented draft versions of the manuscript. AB provided critical comments on the analysis and the manuscript. JPM designed the GLOBE study and provided critical comments on the manuscript. FJvL developed the research questions, supervised the data-analysis and critically commented draft versions of the manuscript. All authors read and approved the final manuscript.

## Supplementary Material

Additional file 1 **Table S1: Measurement of individual cognitions in the GLOBE postal survey 2004**. Click here for file
