# Sports participation, perceived neighborhood safety, and individual cognitions: how do they interact?

**DOI:** 10.1186/1479-5868-8-114

**Published:** 2011-10-20

**Authors:** Mariëlle A Beenackers, Carlijn BM Kamphuis, Alex Burdorf, Johan P Mackenbach, Frank J van Lenthe

**Affiliations:** 1 Department of Public Health, Erasmus University Medical Center, PO Box 2040, 3000 CA Rotterdam, the Netherlands

## Abstract

After publication of this work [Beenackers et al: Int J Behav Nutr Phys Act 2011, 8:76] it was realized that formula 3 and formula 4 in the Statistical Analysis section of the Methods were incorrectly listed. Since the formulas were correctly used in the analysis, this correction does not affect the results or conclusions of the paper.

## Correction

After publication of this work [1] it was realized that formula 3 and formula 4 in the Statistical Analysis section of the Methods were incorrectly listed. Since the formulas were correctly used in the analysis, this correction does not affect the results or conclusions of the paper. The formulas should be:

(3) $$\beta_{1\text{\_}conditional\text{\_}on\text{\_}Z_{medium}}\;\;\;=\beta_{1}+\beta_{6}$$

(4) $$\beta_{1\text{\_}conditional\text{\_}on\text{\_}Z_{low}}\;\;\;=\beta_{1}+\beta_{7}$$

So, to obtain the coefficient of the individual cognition (X) for the second category of perceived neighborhood safety (Z_medium_), the coefficient of X (β_1_) should be added to the coefficient of the interaction term XZ_medium _(β_6_) (equation 3). Because of the logarithmic scale, the odds ratio of an interaction term can be interpreted as a multiplicative factor. To obtain the odds ratio of the individual cognition (X) for the second category of perceived neighborhood safety (Z_medium_), the odds ratio of X (EXPβ_1_) should be multiplied by to the odds ratio of the interaction term XZ_medium _(EXPβ_6_).

To obtain the coefficient of the individual cognition (X) for the last category of perceived neighborhood safety (Z_low_), the coefficient of X (β_1_) should be added to the coefficient of the interaction term XZ_low _(β_7_) (equation 4). Again, to obtain the odds ratio of the individual cognition (X) for the last category of perceived neighborhood safety (Z_low_), the odds ratio of X (EXPβ_1_) should be multiplied by to the odds ratio of the interaction term XZ_low _(EXPβ_7_).

We regret any inconvenience that this may have caused.
