# Supplementary material for: Sports participation, perceived neighborhood safety, and individual cognitions: how do they interact?
Source: Int J Behav Nutr Phys Act. 2011 Jul 21;8:76. doi: 10.1186/1479-5868-8-76 (PMC3197469; doi:10.1186/1479-5868-8-76)
Supplement: Additional file 1 — Table S1: Measurement of individual cognitions in the GLOBE postal survey 2004. [file 1479-5868-8-76-S1.DOC]

# Additional File

Table S1: Measurement of individual cognitions in the GLOBE postal survey 2004

| **Variable** | **Item** | **Response categories** |
| --- | --- | --- |
| **Attitude** | Negative outcome expectancy of physical activity: It requires too much time | (1)Very important – – – (5)Not important at all |
|  | Negative outcome expectancy of physical activity: It requires too much discipline | (1)Very important – – – (5)Not important at all |
|  | Negative outcome expectancy of physical activity: It requires too much energy | (1)Very important – – – (5)Not important at all |
|  | Negative outcome expectancy of physical activity: I am afraid to get injured | (1)Very important – – – (5)Not important at all |
|  | Negative outcome expectancy of physical activity: I feel uncomfortable when exercising | (1)Very important – – – (5)Not important at all |
|  | Positive outcome expectancy of physical activity: It makes me feel less stressed | (1)Not important at all – – – (5)Very important |
|  | Positive outcome expectancy of physical activity: It gets me into a good mood | (1)Not important at all – – – (5)Very important |
|  | Positive outcome expectancy of physical activity: I like being active | (1)Not important at all – – – (5)Very important |
|  | Positive outcome expectancy of physical activity: I am more confident with my body | (1)Not important at all – – – (5)Very important |
|  | Positive outcome expectancy of physical activity: It is good for fitness/condition | (1)Not important at all – – – (5)Very important |
|  | Positive outcome expectancy of physical activity: I feel energized | (1)Not important at all – – – (5)Very important |
| **Social influence** | Most people who are important to me think I should be sufficiently physically active ^a^ | (1)No, not true – (3)Yes, true |
|  | Most people who are important to me stimulate me to be sufficiently physically active | (1)No, not true – (3)Yes, true |
|  | Most people who are important to me are sufficiently physically active | (1)No, not true – (3)Yes, true |
| **Self-efficacy** | Do you think it is easy or difficult to be sufficiently physically active? | (1)Very difficult – – – (5)Very easy |
|  | How sure are you that you can be sufficiently physically active? | (1)Not sure at all – – – (5)Very sure |
| **Intention** | Do you plan to be sufficiently physically active? | (1)No, for sure not – – – (5)Yes, for sure |

^a^ Sufficient physical activity was defined in the questionnaire as being active for at least half an hour a day (e.g. gardening, sports participation, bicycling)
